# Angiosarcoma Revisited: Diagnostic Challenges and a 16-Year Retrospective Analysis from a Single Institution

**DOI:** 10.30699/ijp.2025.2056760.3434

**Published:** 2025-08-15

**Authors:** Swathi Prabhu, Nischitha Suvarna, Kanthilatha Pai, Ranjini Kudva, Deepak Nayak, Harshavardhan Shetty, Vidya Monappa

**Affiliations:** 1 Division of Oncopathology, Department of Pathology, Kasturba Medical College, Manipal, Manipal Academy of Higher Education, Manipal, India; 2 Department of Pathology, Kasturba Medical College, Manipal, Manipal Academy of Higher Education, Manipal, India; 3 Department of Plastic Surgery, Kasturba Medical College, Manipal, Manipal Academy of Higher Education, Manipal

**Keywords:** Angiosarcoma; Diagnosis; Immunohistochemistry; Vascular neoplasms; Histopathology

## Abstract

**Background & Objective:**

Angiosarcomas (AS) are rare, aggressive malignant tumors characterized by marked histopathologic heterogeneity, often mimicking other neoplasms and complicating diagnosis. This 16-year retrospective study aimed to evaluate the clinicopathological spectrum of AS, with particular emphasis on diagnostic challenges and strategies for accurate identification.

**Methods:**

We retrospectively reviewed 11 histologically confirmed cases of AS diagnosed at our institution between January 2008 and December 2023. The data collected included patient demographics, clinical presentation, tumor location, histopathologic features, immunohistochemical (IHC) profiles, treatment modalities, and clinical outcomes.

**Results:**

Patient ages ranged from 25 to 62 years, with a slight female predominance (male-to-female ratio, 0.8:1). Tumor locations were variable, and histologic patterns ranged from well-differentiated, low-grade vascular proliferations resembling hemangiomas to poorly differentiated neoplasms mimicking pleomorphic undifferentiated sarcomas. IHC findings demonstrated overlapping marker expression, including cytokeratin (CK) positivity, which may lead to misdiagnosis as carcinoma, and loss of H3K27me3 expression, which can raise suspicion for malignant peripheral nerve sheath tumors (MPNST). Several cases also exhibited morphologic features closely resembling epithelioid hemangioendothelioma.

**Conclusion:**

Angiosarcoma poses considerable diagnostic difficulty due to its morphologic variability and overlapping immunophenotypic profiles. Accurate diagnosis necessitates a multidisciplinary approach, integrating clinical, radiologic, and pathologic data. This study highlights the importance of diagnostic vigilance and comprehensive evaluation when assessing vascular neoplasms.

## Introduction

Angiosarcomas (AS) are rare and aggressive malignant tumors that account for approximately 1% of all soft tissue sarcomas. They replicate endothelial cells both morphologically and immunohistochemically (1). AS may arise de novo; however, established risk factors include chronic lymphedema—particularly following breast surgery—and prior radiation exposure. Approximately 60% of cases occur in cutaneous regions, most commonly involving the head, neck, and upper extremities, including the scalp. The remaining cases occur in deeper tissues such as the viscera, bones, and other internal organs (2). AS may also develop in association with implants (eg, following breast cancer surgery), within preexisting hemangiomas or vascular malformations, or, in rare cases, as part of syndromic conditions.

AS is a highly aggressive and infiltrative tumor, evidenced by its high rates of local recurrence and distant metastasis (1,3). Unlike other sarcomas, AS generally exhibits low frequencies of alterations in the TP53 and PIK3CA/AKT/mTOR pathways (4). Instead, the upregulated genes are commonly associated with angiogenesis and vascular-specific receptor tyrosine kinases, such as TIE1, TEK, KDR (VEGFR2), and FLT4 (VEGFR3) (5,6). In post-irradiation or chronic lymphedema-associated cases, high-level MYC gene amplification is often observed and can be demonstrated using MYC immunohistochemistry (7). AS shows a male predominance and typically presents in the seventh decade of life (8).

This study of 11 histopathologically confirmed AS cases highlights their rare and unusual presentations. The aim of this study is to comprehensively analyze the clinicopathological features of AS, with a particular focus on diagnostic challenges and the methods used to overcome them.

## Materials and Methods

### Study Design

A retrospective analysis was conducted in the Department of Pathology at our institution using the hospital's electronic medical records (EMR) system. The study included patients with histopathologically confirmed diagnoses of AS between January 2008 and December 2023.

### Inclusion CriteriaPatients with a histopathologically confirmed diagnosis of AS during the study periodAvailability of complete clinical, radiologic, and histopathologic data

### Exclusion CriteriaCases in which histologic slides were unavailable for review

### Data Collection

Data were extracted from pathology reports, patient medical records, and imaging studies. Variables collected included patient demographics, clinical presentation, tumor location and size, histopathologic features, treatment modalities, and clinical outcomes.

### Histopathological Examination

All available tissue samples were independently re-evaluated by two experienced pathologists to confirm the diagnosis.

In total, 11 cases were identified and analyzed over the 16-year study period. All relevant data were retrieved and included in the final analysis.

## Results

Patient ages ranged from 24 to 62 years, with a mean age at diagnosis of 50 years. The male-to-female ratio was 0.8:1. Histomorphological features ranged from well-differentiated vascular channels to poorly differentiated, high-grade forms. One case uniquely demonstrated Verocay bodies. Necrosis was observed in 8 of 11 cases (72.7%), lymphovascular invasion (LVI) in 2 cases (18.2%), and perineural invasion (PNI) in 1 case (9%).

**Fig 1:**
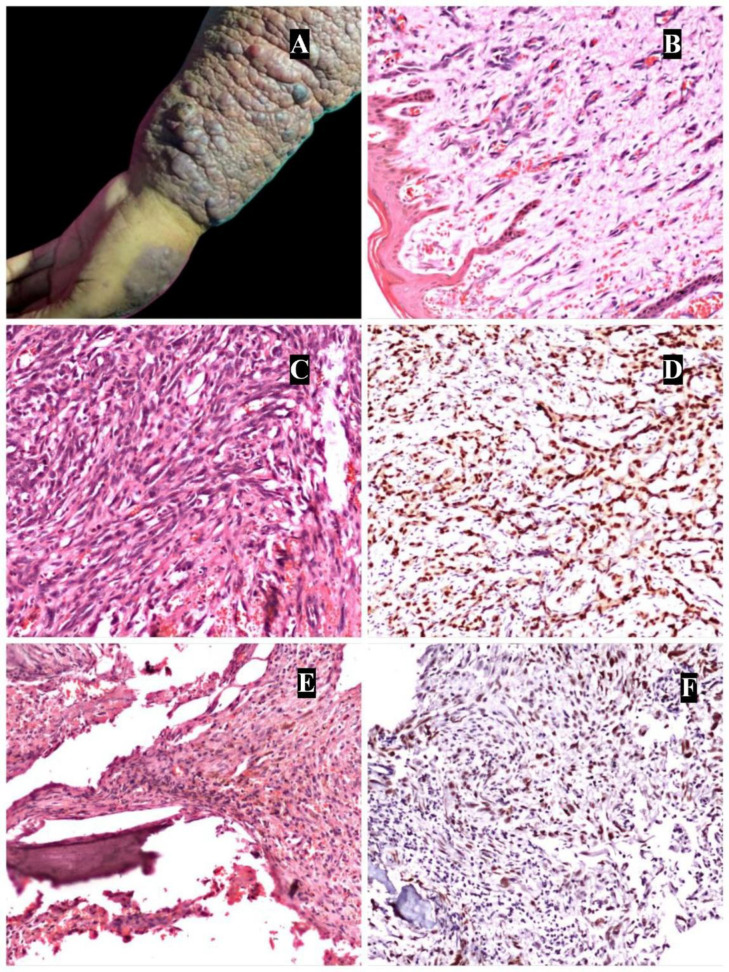
Clinical photograph of angiosarcoma in a patient previously treated for breast carcinoma. A superficial initial biopsy revealed angiosarcoma with well-formed capillary channels, which could be mistaken for hemangioma. However, deeper sections from the resection specimen demonstrated poorly differentiated, high-grade areas (Figure 1B, C). Figure 1D highlights ERG positivity in this neoplasm, confirming the vascular origin. Figure 1E shows angiosarcoma involving the vertebrae, with ERG-positive tumor cells (Figure 1F).

**Fig 2:**
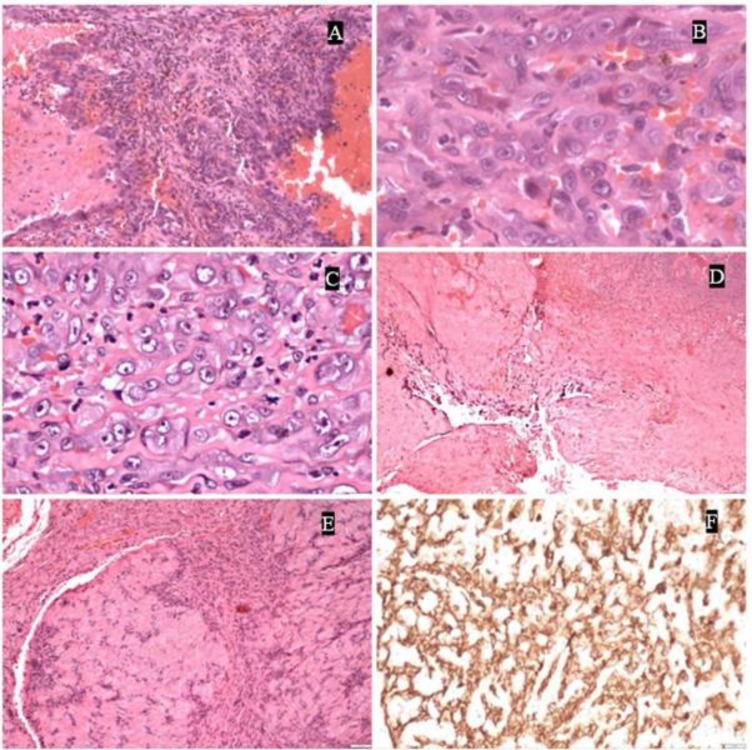
Image highlights the morphological diversity observed in angiosarcomas. Figure 2A shows areas with large vascular spaces interspersed with solid regions demonstrating vascular channel proliferation. Figure 2B depicts poorly differentiated angiosarcoma with minimal vascular differentiation, predominantly consisting of solid tumor areas (as seen in Figure 2C). Figure 2D illustrates large vascular spaces lined by atypical cells. NotablyFigure 2E demonstrates the presence of verrocay bodies, a feature typically associated with schwannoma, but immunohistochemistry with ERG (Figure 2F) confirms the diagnosis as angiosarcoma.

Tumor sites were diverse. Notably, one case of colonic AS presented as polypoid lesions, while another arose in bone, and one was associated with prior radiation exposure (Figures 1 and 2). Additional sites of involvement included the mediastinum, oral cavity, scalp, and breasts. Metastasis was documented in 3 cases (27.3%), with the lungs, vertebrae, and lymph nodes being the most commonly affected sites.

Table 1 provides a comprehensive summary of patient age, sex distribution, tumor site, histopathologic features, immunohistochemical findings, treatment modalities, and clinical outcomes.

### Immunohistochemistry

Cytokeratin (CK) positivity was detected in 66.6% of cases. Endothelial markers ERG and FLI1 were positive in all cases. CD34 positivity was observed in 90% of cases. Notably, concurrent loss of H3K27me3 expression and CMYC amplification was identified in a single case of post-radiation-induced AS. Table 2 summarizes the immunohistochemical profiles of the cases.

### Survival

In our study, patient survival varied significantly. One patient died within 3 days of diagnosis, following surgery, while another survived for 2 years. Most of our patients, however, succumbed to the disease within a year of diagnosis, highlighting the aggressive nature of AS. Survival analysis could not be performed because of the small number of cases. 

**Table 1:** presents detailed information on all 11 cases in our study, encompassing patient age, clinical history, immunohistochemical (IHC) marker variations, treatments provided, and follow-up data.

Age/sex	Site	Diagnosis	Morphological findings	IHC	Treatment	Follow up
Case 1 60-years female In known case of carcinoma breast (7 years back)	Left upper limb	Post irradiation/Chronic lymphedema associated angiosarcoma	Anastomosing thin-walled vascular channels Necrosis + LVI* - PNI **+	ERG, CD34, FLI-1, CMYC, CK positive H3K27me3 loss	Left upper limb disarticulation	Tumour recurred at the site after 5 months with axillary lymph node metastasis. Patient under follow up
Case 2 55-years male In a known case of carcinoma tongue (8 years back)	At the PMMC flap site	Angiosarcoma	Large vascular spaces lined by malignant tumour cells with solid sheets of cells Necrosis+ LVI – PNI-	Vimentin, FLI-1, ERG positive SMA, CK, CD31 patchy positive P40, CD34, S100, SOX10 negative H3K27me3, INI1 retained CMYC negative	Chemotherapy	Patient died 6 months after the diagnosis of angiosarcoma
Case 3 45-years male	Base of penis	Epithelioid angiosarcoma	Anastomosing thin-walled vascular channels Pseudopapillary pattern, verocay bodies Necrosis- LVI – PNI-	CK, CD34, FLI-1 positive SMA patchy expression H3K27me3, INI1 retained	Palliative radio and chemotherapy	Patient died 14 months following initial diagnosis
Case 4 61-years female	Mediastinum	Angiosarcoma	Anastomosing thin-walled vascular channels Necrosis – LVI- PNI-	CK, ERG, CD34, FLI-1 positive	Chemotherapy	No follow up available
Case 5 53-years female	Multiple organs (Lungs, lytic lesions of bone, breast)	Initially diagnosed as epithelioid haemangioendothelioma. Reviewed outside as Disseminated epithelioid angiosarcoma	Nests and cords of cells with moderate atypia Necrosis- LVI- PNI-	CD34, ERG positive Synaptophysin, CK, GATA3, S100, Desmin negative	Chemotherapy and radiotherpy	Patient died two years two months following diagnosis
Case 6 54-years female	Liver disseminated to spleen vertebrae	Angiosarcoma	Nests and fascicles, areas of anastomosing vascular channels Necrosis+ LVI- PNI-	ERG, FLI-1, CD34, Vimentin positive LCA, CD21, CD68, Arginase negative	NA^$^	No follow up
Case 7 59-years female In a known case of carcinoma cervix, post hysterectomy, post chemoradiotherapy (12 years back)	Right leg	Angiosarcoma	Sheets and nests of anastomosing vascular channels Necrosis+ LVI- PNI-	CD34 positive, D240 positive CK, HMB45, P63 negative	NA	Discharged against medical advice
Case 8 24-years female Lactating	Right breast with lung and lymph node metastasis in lactating woman	Angiosarcoma	Arborising vascular channels Necrosis+ LVI+ PNI-	CD34 positive ER, PR, HER2neu negative	Chemotherapy	Died 6 months following initial diagnosis
Case 9 62-years male	Bone	Epithelioid angiosarcoma	Anastomosing vascular channels and cystic spaces lined by tumour cells Necrosis+ LVI- PNI-	CD34 positive	Surgery followed by post operative chemotherapy	Died one month following initial diagnosis
Case 10 51-years male	Colonic disseminated epithelioid angiosarcoma presenting as multiple intestinal polyps	Disseminated epithelioid angiosarcoma	Multiple polypoidal projections composed of anatomising vascular channels of atypical cells Necrosis+ LVI+ PNI-	CD34 positive	Colectomy	Died three days following surgery
Case 11 35-years male	Skull	Angiosarcoma	Anastomosing thin-walled vascular channels Necrosis+ LVI- PNI-	CD34, FLI-1 positive	Surgery	Diagnosed as giant cell tumour else where and was treated accordingly. The case was reviewed later, and patient died three months later

*LVI: Lymphovascular invasion **PNI: Perineural invasion $ NA: Not applicable

**Table 2:** The table depicts the percentage of cases showing positivity for various IHC markers

Immunohistochemistry markers	Percentage of cases showing positivity (N=11) %
CK	66.6%
ERG	100%
FLI-1	100%
CMYC	20%
CD34	90%
p53	No mutant type staining noted; Positivity ranged from 1-3%

## Discussion

Angiosarcomas (AS) are rare sarcomas that can occur in virtually any part of the body. These tumors carry a poor prognosis, characterized by high rates of local recurrence and a tendency for lymph node metastasis, with overall survival ranging from 6 to 16 months (9). They demonstrate a male predominance and typically affect individuals in their seventh decade of life. AS is extremely rare in children. Clinically, these tumors are often rapidly growing, painful, poorly defined, and may present with lymph node involvement or distant metastasis (10). In our case series, the youngest patient was a 25-year-old lactating woman and the oldest a 62-year-old man. Interestingly, we observed a slight female predominance (male-to-female ratio, 0.8:1), contrasting with literature reports. This reversal likely reflects the small sample size of our study.

AS most commonly arises in the head and neck region, especially the scalp, but it can develop anywhere in the body (11). In our study, tumor locations were varied. Two cases involved the head and neck, while three cutaneous cases affected the upper limb, base of the penis, and lower limb. The remaining cases involved visceral sites. Additionally, one case of osseous AS was previously published as a case report (12). Three patients (27.2%) had a history of prior malignancy and radiotherapy. One case represented classic Stewart–Treves syndrome, occurring after breast cancer surgery. Another patient, with a history of oral cavity carcinoma, developed AS at the pectoralis major myocutaneous (PMMC) flap site. A third case involved a patient with cervical carcinoma. Notably, one patient presented with multiple polypoidal lesions in the small intestine and colon. Despite undergoing colectomy, the patient died three days postoperatively. AS involving the gastrointestinal tract is rare, most commonly affecting the stomach or small intestine. Colonic AS is particularly uncommon, with only approximately 40 cases reported globally (13).

Kindblom et al. reported local recurrence in 20% and distant metastasis in 49% of 80 AS cases, with the lungs being the most frequent metastatic site, followed by lymph nodes (10). In our cohort, distant metastases were observed in 3 patients (27.2%). One case exhibited metastases to both the bone and lung, another to the lung alone, and a third to the spleen and bone.

Histomorphologically, AS typically demonstrates anastomosing vascular channels with cytologic atypia and necrosis. However, well-differentiated or low-grade AS can mimic hemangioma, particularly when the clinical and radiological context is not considered. AS is notorious for its histological heterogeneity, with patterns ranging from well- to poorly differentiated, often lacking identifiable vascular architecture (10). In our first case, the biopsy revealed well-formed vascular channels with minimal atypia, potentially suggestive of hemangioma. However, the clinical background—multiple purple nodules in the arm of a breast cancer survivor—suggested Stewart–Treves syndrome. Examination of the amputation specimen confirmed high-grade features, including marked atypia and necrosis, underscoring the importance of clinicopathological correlation.

AS must be distinguished from histologic mimics such as hemangioma and Kaposi sarcoma (KS). Hemangiomas are generally superficial and exhibit minimal atypia, with rare mitoses. The anastomosing hemangioma variant may mimic AS (14–16), but lacks the infiltrative growth seen in AS. Immunohistochemistry (IHC) may not always help, as both express CD34 and ERG. However, Ki-67 proliferation index is typically low in hemangiomas and high in AS, reflecting aggressive behavior (16).

Spindle cell AS may mimic KS, a locally aggressive tumor associated with human herpesvirus 8 (HHV-8), particularly in immunocompromised individuals. KS progresses through patch, plaque, and tumor phases (17), and immunohistochemically expresses CD34, ERG, and D2-40, with variable CD31 expression (18). Nuclear positivity for HHV-8 LANA-1 is pathognomonic and essential for definitive diagnosis (17).

One of our cases displayed alternating hypo- and hypercellular areas resembling a schwannoma, raising concern for malignant peripheral nerve sheath tumor (MPNST). However, extensive sampling revealed classic AS morphology. Retained H3K27me3 expression on IHC helped exclude MPNST. While rare, AS arising within schwannomas has been reported (19–21), but our patient had no such history. CD34 positivity supported a vascular origin. This case highlights the importance of thorough sampling and judicious use of IHC in diagnostically challenging cases.

Deyrup et al. proposed a prognostic model for cutaneous AS, classifying tumors as high- or low-risk based on necrosis and epithelioid morphology. High-risk tumors had a 3-year survival rate of 24% versus 77% in the low-risk group (22). Applying this model to our study, all but one case were classified as high-risk. However, survival outcomes within this group varied, from 3 days postoperatively to 2 years, suggesting that this stratification may not be applicable to visceral AS.

Epithelioid hemangioendothelioma (EHE) usually does not pose a diagnostic dilemma unless it displays high-grade features such as nuclear atypia, necrosis, high mitotic rate, or solid areas, which occur in ~10% of cases (23). In our series, one case (case 5) was initially diagnosed as EHE but was later revised to AS by a reference oncopathology lab, based on CAMTA negativity. The patient survived for 2 years post-diagnosis. The absence of CAMTA or TFE3 IHC likely contributed to the initial diagnostic uncertainty. In this case, the revised diagnosis did not significantly alter clinical management.

H3K27me3 loss is a well-established marker for MPNST, but it can be seen in other sarcomas, particularly those induced by radiation (24–26). One radiation-associated case in our study (case 1) showed H3K27me3 loss, while another did not, highlighting marker variability and the need for a comprehensive diagnostic approach.

ERG, a transcription factor of the ETS family, is a sensitive marker for vascular tumors, but it can be expressed in other malignancies, such as epithelioid sarcoma, Ewing sarcoma, and prostate adenocarcinoma (27). Therefore, additional markers like CD31 and FLI-1 may aid diagnosis, though they also lack absolute specificity (28). CD31 is more sensitive than CD34 and was widely used before ERG became standard (29).

Cytokeratin (CK), an epithelial marker, may be focally positive in AS, especially in poorly differentiated forms. In our series, CK was positive in 66.6% of cases, though the staining was focal to moderate, not diffuse. One colonic AS case with CK positivity could have been mistaken for adenocarcinoma, but hemorrhagic features prompted further IHC, confirming AS. Abbadi et al. reported CK positivity in 3% and EMA positivity in 10% of AS cases (31). CK positivity in AS with lymph node metastasis can lead to misdiagnosis as carcinoma unless carefully evaluated in clinical context.

p53 IHC was available in five of our cases and showed low expression (1%–3%), offering limited diagnostic utility. Nonetheless, literature suggests that p53 overexpression may occur in some AS, implicating it in tumorigenesis (32).

AS remains a highly aggressive malignancy with poor survival outcomes. Buehler et al. reported a 5-year survival rate of 40%, with a median survival of 16 months. Poor prognostic indicators include visceral or deep soft tissue location, size >5 cm, necrosis, metastasis at diagnosis, and incomplete surgical excision (9). Smrke et al. emphasized the importance of complete surgical resection, with radiotherapy and chemotherapy also contributing to improved survival (33). Emerging evidence suggests that PD-L1 expression may predict immunotherapy response in cutaneous AS (34). A systematic review by Shin et al. identified favorable prognostic factors in AS of the face and scalp, including age <70 years, tumor size <5 cm, facial location, and surgical resection (35).

To summarize, AS is a rare, aggressive malignancy with highly variable clinical and histopathological presentations. Diagnostic challenges arise due to its morphologic heterogeneity and overlapping immunophenotypes with other vascular and non-vascular tumors. Comprehensive pathologic evaluation, including careful sampling and an extended IHC panel, is essential to avoid misdiagnosis. While risk stratification models exist, their application to non-cutaneous AS may be limited. Our findings underscore the need for individualized diagnostic strategies and treatment approaches. Larger studies with long-term follow-up are warranted to better characterize prognostic factors and improve patient outcomes.

## Conclusions

This study highlights the diagnostic complexity of angiosarcoma, driven by its variable morphology and immunohistochemical profiles. A comprehensive pathological evaluation using multiple vascular markers is essential to avoid misdiagnosis. Despite the small sample size, our findings underscore the broad clinical and histological spectrum of AS and emphasize the need for an integrated clinicopathologic and radiologic approach to ensure accurate diagnosis and effective management of this aggressive malignancy.

## Data Availability

There is no additional data separate from available in cited references.
